# Insulinoma in Patient With Nonalcoholic Steatohepatitis

**DOI:** 10.7759/cureus.43469

**Published:** 2023-08-14

**Authors:** Daniel Cain, Ricardo Anguiano-Albarran, Franklin Obi, Sidart Pradeep, Steven Mudrovich, Melvin Simien

**Affiliations:** 1 Internal Medicine, Baylor Scott & White All Saints Medical Center, Fort Worth, USA; 2 Pathology, Baylor Scott & White All Saints Medical Center, Fort Worth, USA; 3 Gastroenterology and Hepatology, Baylor Scott & White All Saints Medical Center, Fort Worth, USA; 4 Interventional Endoscopy, Baylor Scott & White Digestive Diseases, Fort Worth, USA

**Keywords:** histopathology, gastroenterology and endoscopy, insulinoma, eus, nash, pancreatic insulinoma

## Abstract

An insulinoma is a rare neuroendocrine tumor characterized by inappropriate secretion of insulin with resultant hypoglycemia and concomitant symptoms. Symptoms include diaphoresis, tremor, palpitations, tachycardia, visual disturbances, weakness, confusion, syncope, seizures, and even coma. Enteropancreatic neoplasms are rare in general but among them, insulinomas are among the more common neuroendocrine tumors though they still have a very low incidence. They can be benign or malignant, however, the latter is exceptionally rare. In the case of malignancy, such spread usually includes metastasis to the liver and surrounding nodes. They can also be sporadic or occur in association with other inherited conditions. Herein, we present a case of insulinoma in a 51-year-old female.

## Introduction

Insulinomas are a rare pancreatic neuroendocrine tumor (pNET) that when functional, releases endogenous insulin with no regard for the body’s normal regulating mechanisms. Patients present with symptoms of hypoglycemia including diaphoresis, tremor, cardiac palpitations, visual changes, weakness, altered mentation, syncope, seizure, as well as coma. The estimated incidence of insulinomas is four per one million people in the US annually [1]. Sporadic insulinomas are more common than those associated with inherited genetic conditions, accounting for 92%-95% of all insulinomas [2]. They are typically under 2 cm in size and can be located throughout the pancreas [1]. 

There is a strong association between pNETs and multiple endocrine neoplasia type 1 (MEN-1), with 30%-80% of MEN-1 patients having a pNET, and 10% of all pNETs occurring in MEN-1 patients [3]. Insulinomas are the second most common form of pNET seen in MEN-1 patients accounting for 10%-30% of pNETs in this population [4]. Insulinomas are also likely to occur at ages younger than 50 (many in their twenties) and are larger in size when in a MEN-1 patient [2,3,5]. 

Insulinomas are primarily solitary tumors but have been known to become metastatic. In a study by Sada et al., the incidence of malignancy between sporadic and MEN-1-associated tumors was not found to be statistically significant amongst their 311 patient cohort, with 18% and 16% being reported, respectively. Recurrence of insulinomas was also noted to be high in MEN-1 patients in this study [5]. 

Diagnosis is started with strong clinical suspicion based on the presentation and symptoms listed above. Workup is commonly delayed by ruling out non-pNET causes for the various symptoms that a patient can present with. Whipple’s triad criteria have been used to diagnose insulinomas historically. The three elements are episodic hypoglycemia with plasma glucose <50 mg/dL, mental status changes related to hypoglycemia, and reversal of symptoms upon correction of plasma glucose. A 72-hour fast with testing of insulin and blood glucose should also be completed as it is the gold standard of testing [6-8]. Hypoglycemia evaluation should be carried out for any evidence of exogenous versus endogenous insulin. Insulin, pro-insulin, C-peptide levels, and plasma glucose during a 72-hour fast have been found to have 99% sensitivity [9]. Labs indicative of insulinoma during this fasting period are hypoglycemia with elevations in plasma insulin level of ≥6 μU/ml, C peptide level ≥0.2 nmol/l, and proinsulin level ≥22 pmol/l [10,11]. Insulin antibody levels should also be checked in case of any autoimmune etiology. Recently, serum assays testing for insulin and proinsulin have been developed and found to be 90%-95% sensitive for insulinoma when paired with a 48-hour fast [10]. 

Once the above criteria and lab values have been obtained, imaging for localization and further evaluation is warranted. Multidetector helical CT is the preferred initial imaging modality due to its high sensitivity of 83%-94.4% [12]. The thin slicing and use of contrast at various stages of the CT scan with subsequent endoscopic ultrasound (EUS) and fine needle aspiration (FNA) provides the most information on location and lesion contents to guide any possible surgical treatment. In a meta-analysis on EUS success in detecting neuroendocrine tumors by Puli et al., EUS demonstrated a pooled sensitivity of 87.5% and a pooled specificity of 97.4% for insulinomas (nine studies representing 242 patients) [13]. Localized treatments, ranging from chemotherapy to surgical, can be evaluated based on further staging and other patient factors.

## Case presentation

We present a case of a 50-year-old female with a past medical history of hypothyroidism, anemia, and migraines. She presented within 24 hours of abdominal pain, which was characterized as constant burning epigastric pain that was non-radiating. She had associated nausea and diarrhea since symptom onset. She denied any recreational substance use, alcohol, or tobacco use. Her family history was significant for diabetes mellitus on her mother’s side. Surgical history was significant for previous cholecystectomy; liver function tests (LFTs), complete metabolic panel (CMP), complete blood count (CBC), and lipase were all within normal limits. Her physical exam was significant for mild epigastric pain. Ultrasound of the abdomen in the emergency department showed mild nodular surface changes. CT abdomen was negative aside from incidental right renal angiomyolipoma. She was discharged with a diagnosis of duodenitis and gastritis, and prescribed ondansetron and pantoprazole.

**Figure 1:**
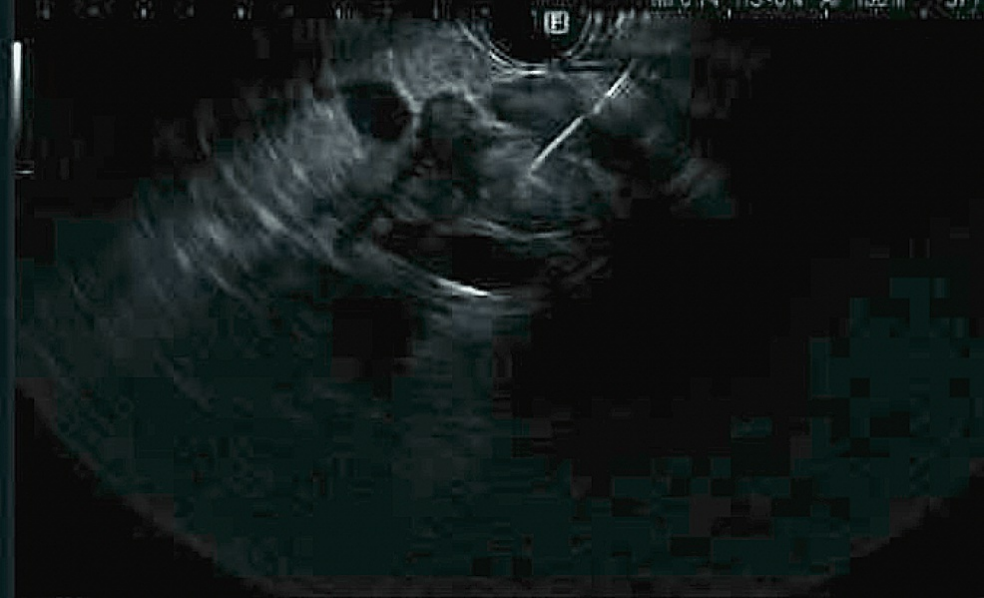
Endoscopic ultrasonography (EUS) imaging with fine needle aspiration (FNA) of the pancreas

**Figure 2:**
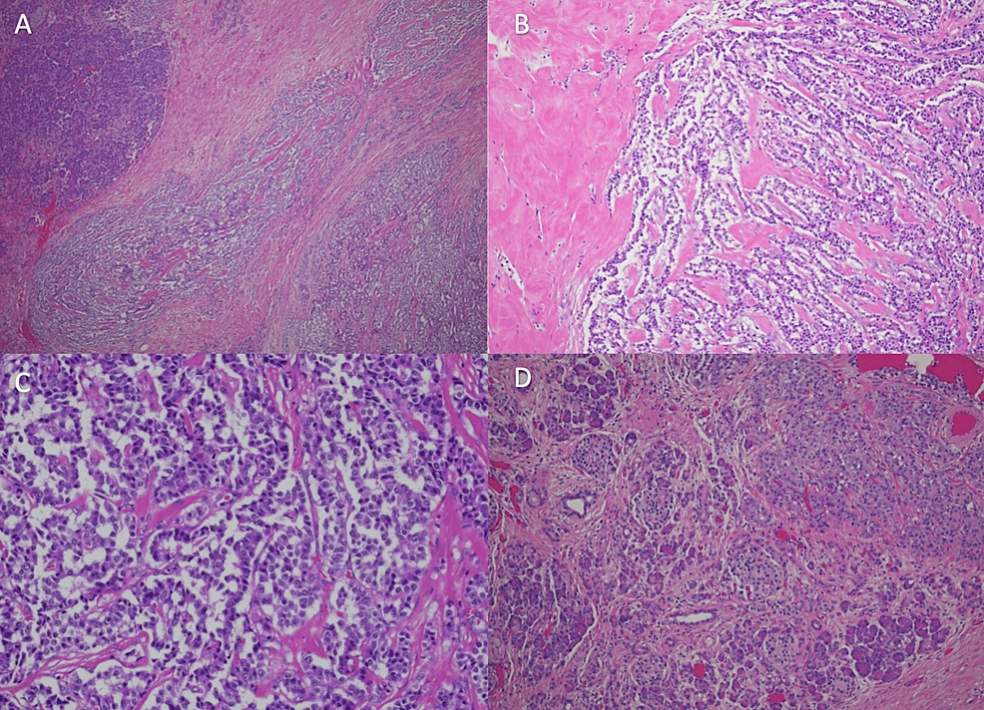
H&E staining of endoscopic ultrasonography (EUS) pancreatic insulinoma sample A) Low power H&E stain; B) Medium power H&E stain with central sclerosis; C) High power H&E stain of neuroendocrine tumor of the pancreas; D) Tumor cells with adjacent pancreas islets.

**Figure 3:**
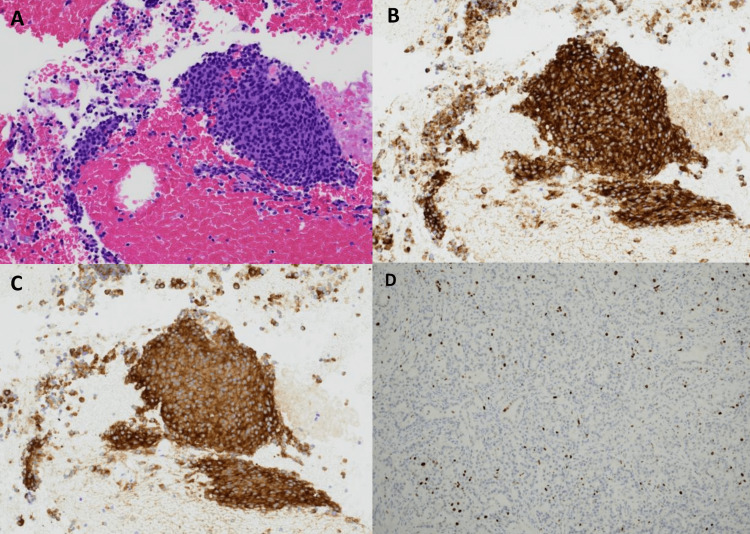
Specialized staining of the endoscopic ultrasonography (EUS)/fine needle aspiration (FNA) sample of the pancreatic insulinoma A) High power H&E stain; B) Positive chromogranin stain; C) Positive synaptophysin stain; D) Ki-67 staining

Outpatient follow-up after the emergency room visit revealed the patient had been having intermittent epigastric pain prior to presentation, with flares that would radiate to her back. She also had been having a globus sensation and vomiting intermittently in the morning for about seven months. She also reported concern about her increase in weight without significant lifestyle or dietary changes. Her baseline weight was 130 pounds and she was now 201 pounds. She did report pantoprazole was improving her symptoms, and she was scheduled for a follow-up esophagogastroduodenoscopy (EGD) to evaluate for ulcerations. She was also scheduled for endoscopic ultrasonography (EUS) with plans for a liver biopsy due to the difference between the ultrasound and CT scan done at the emergency room. EGD was significant for diffuse gastritis with ulcerations, and a Hill grade IV hiatal hernia. During the EUS, the pancreas was visualized, and a 27 x 17 mm mass was noted in the tail of the pancreas. A fine needle biopsy was performed at the pancreatic lesion and liver for further evaluation (Figures 1-3). Insulin level was found to be 226.3 uIU/mL (reference range, 19.6 uIU/mL) and C-peptide was 14.77 ng/mL (reference range 0.80-3.85 ng/mL), consistent with insulinoma. EUS results showed nonalcoholic steatohepatitis grade 1, with an area of focal necrosis for the liver biopsy. The pancreatic mass was positive for synaptophysin, chromogranin, and cytokeratin CAM 5.2 staining. The Ki-67 was less than 3%, though pathology noted this was due to the wide disbursement of the tissue in the prepared sample and may not be truly representative of the lesion.

**Figure 4:**
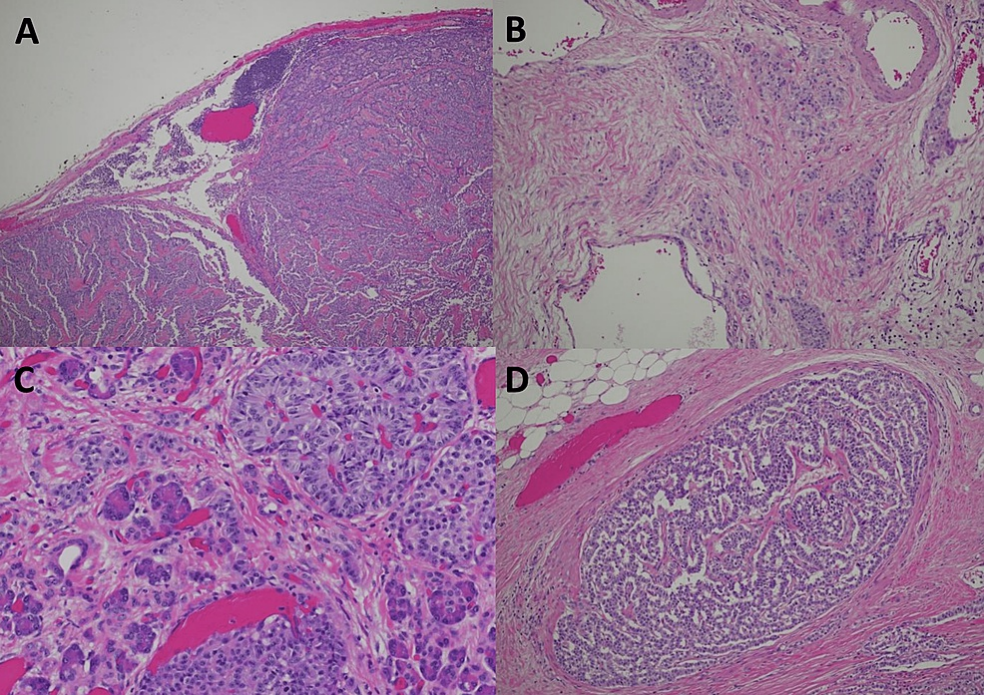
Staining of the insulinoma surgical specimen A) Metastasis involving nearly the entire lymph node; B) Diffuse and multinodular tumor invasion at high power; C) Diffuse extrapancreatic invasion; D) Tumor invasion of the large vein with smooth muscle wall

The patient was then referred to oncology and surgical oncology for further evaluation and she was treated with distal pancreatectomy and splenectomy with concomitant hiatal hernia repair. The pancreatic mass was sent to pathology for further evaluation and measured 5.1 cm, with two of two pancreatic lymph nodes positive for metastatic neuroendocrine tumor. Histopathological evaluation of the tumor was conducted and was positive for synaptophysin, chromogranin, and cytokeratin CAM 5.2 (Figure 3). Ki-67 was repeated as well and estimated to be 15%. The overall features are compatible with a WHO grade 2 tumor. Further evaluation with CT abdomen and pelvis with contrast showed a stable lesion in hepatic segment 8, with a recommendation for MRI with contrast for further evaluation if needed (Figure 4). As of now, the patient has completed recovery after surgical resection of the mass, and a follow-up PET scan showed mild enhancement of a left crural lymph node. She underwent a surgical biopsy of this node which was found to be benign with no abnormal cellularity or architecture. She is continuing follow-up and ongoing surveillance imaging is being conducted with her oncologist. 

## Discussion

The case presented above is characteristic of an incidental insulinoma that was seen and biopsied during the EUS. The PET scan with the concerning lymph node did not have available documentation on whether GLP-1 analogues were used for further detection of any metastatic lesions as this type of pNET. The use of these analogies is important since it has been demonstrated in multiple studies to be the preferred method of identifying metastatic insulinomas even if very small in size [14-16]. Luo et al. demonstrated the effectiveness of using 68Ga-DOTA-exendin-4 as the GLP-1 analogue as it showed a 97.7% sensitivity for insulinomas [17]. 

The tumor in our case was determined to be a WHO grade 2 tumor, based on the 2019 criteria (Table 1) [18]. This classification system looks at the mitotic rate of the tumor as well as the Ki-67 index. Both of these measurements are done to gauge the tumor growth but are not always uniformly elevated [19]. Per WHO guidelines, whichever measurement would cause the highest tumor grade should be the measurement used to characterize the tumor. The Ki-67 index in our patient was estimated to be 15% when the full tumor was examined, which led to the WHO Grade 2 classification.

**Table 1:** WHO grading system for neuroendocrine neoplasms (NENs) of the gastrointestinal tract and hepatopancreatobiliary organs NET: neuroendocrine tumor; NEC: necrotizing enterocolitis; MINEN: mixed neuroendocrine-non-neuroendocrine neoplasms

Classification	Differentiation	Grade	Mitotic Rate (Mitoses/2mm^2)	Ki-67 Index
NET, G1	Well Differentiated	Low	<2	<3%
NET,G2	Well Differentiated	Intermediate	2-20	3-20%
NET, G3	Well Differentiated	High	>20	>20%
NEC, Small-Cell Type (SCNEC)	Poorly Differentiated	High	>20	>20%
NEC, Large-Cell Type (LCNEC)	Poorly Differentiated	High	>20	>20%
MiNEN	Variable	Variable	Variable	Variable

Other histological studies used for insulinoma identification are not required for diagnosis, but can further solidify the diagnosis [20]. Chromogranin and synaptophysin staining is utilized to confirm neuroendocrine etiology. Chromogranin is involved in the regulation of secretory granules, and synaptophysin is a protein in the synaptic vesicles [21]. They are non-specific markers for insulinoma but are indicative of neuroendocrine tumors as a whole. Chromogranin CAM 5.2 staining is highly positive in secretory tissues, such as functional pNETs [22]. Our patient was positive for all three on both UES and surgical pathology examination, which would be expected with an insulinoma.

The nonalcoholic steatohepatitis seen on the biopsy of our patient is also not unexpected given the weight changes she experienced secondary to the excess endogenous insulin. She was reported to have had a seventy-pound weight gain and the high level of increased lipogenesis in the setting of the elevated insulin likely contributed to this. This phenomenon has been reported in another insulinoma case by Rokutan et al. In their case, the patient had rapid weight gain and elevated LFT that resolved after the insulinoma was resected [23].

Surgical treatment is the definitive choice for treating insulinomas, once lesion localization has been completed. The utility of chemotherapy is still under investigation as there has not been enough evidence to endorse widespread implementation in the treatment of pNETs [16]. Surgical options are dependent on the involvement of the pancreas and proximity to surrounding structures. For localized lesions, surgical enucleation can be performed. This involves resection of the tumor with thin surrounding margin for small single tumors. If the tumor is larger, distal pancreatectomy or pancreaticoduodenectomy may be indicated [24-25]. There is a 3% recurrence rate after resection, and it has been associated with WHO grade 2 insulinomas and malignancy [25]. MEN-1 patient recurrence is roughly 21% [26]. Splenectomy was performed in our patient in addition to distal pancreatectomy due to the risk for metastasis, though low-grade and benign lesions can be considered for pancreatic preservation [27]. The prognosis post resection is promising with the 10-year survival rate being 88% in non-malignant insulinomas. Malignant insulinomas carry a poorer prognosis with a 10-year survival rate of 29% [26].

## Conclusions

The goal of this report is to contribute to the existing body of literature on this rare cancer. The associated liver findings and presentation with gastritis as a chief complaint serve to show that insulinomas do not always present with the classic hypoglycemic picture. We hope our case adds to the ongoing study of this rare pathology.
